# Intimate Partner Violence and Incident Depressive Symptoms and Suicide Attempts: A Systematic Review of Longitudinal Studies

**DOI:** 10.1371/journal.pmed.1001439

**Published:** 2013-05-07

**Authors:** Karen M. Devries, Joelle Y. Mak, Loraine J. Bacchus, Jennifer C. Child, Gail Falder, Max Petzold, Jill Astbury, Charlotte H. Watts

**Affiliations:** 1 London School of Hygiene & Tropical Medicine, London, United Kingdom; 2 University of Gothenburg, Göteborg, Sweden; 3 Monash University, Clayton, Australia; Massachusetts General Hospital, United States of America

## Abstract

**Background:**

Depression and suicide are responsible for a substantial burden of disease globally. Evidence suggests that intimate partner violence (IPV) experience is associated with increased risk of depression, but also that people with mental disorders are at increased risk of violence. We aimed to investigate the extent to which IPV experience is associated with incident depression and suicide attempts, and vice versa, in both women and men.

**Methods and Findings:**

We conducted a systematic review and meta-analysis of longitudinal studies published before February 1, 2013. More than 22,000 records from 20 databases were searched for studies examining physical and/or sexual intimate partner or dating violence and symptoms of depression, diagnosed major depressive disorder, dysthymia, mild depression, or suicide attempts. Random effects meta-analyses were used to generate pooled odds ratios (ORs). Sixteen studies with 36,163 participants met our inclusion criteria. All studies included female participants; four studies also included male participants. Few controlled for key potential confounders other than demographics. All but one depression study measured only depressive symptoms. For women, there was clear evidence of an association between IPV and incident depressive symptoms, with 12 of 13 studies showing a positive direction of association and 11 reaching statistical significance; pooled OR from six studies = 1.97 (95% CI 1.56–2.48, *I* ^2^ = 50.4%, *p*_heterogeneity_ = 0.073). There was also evidence of an association in the reverse direction between depressive symptoms and incident IPV (pooled OR from four studies = 1.93, 95% CI 1.51–2.48, *I* ^2^ = 0%, *p* = 0.481). IPV was also associated with incident suicide attempts. For men, evidence suggested that IPV was associated with incident depressive symptoms, but there was no clear evidence of an association between IPV and suicide attempts or depression and incident IPV.

**Conclusions:**

In women, IPV was associated with incident depressive symptoms, and depressive symptoms with incident IPV. IPV was associated with incident suicide attempts. In men, few studies were conducted, but evidence suggested IPV was associated with incident depressive symptoms. There was no clear evidence of association with suicide attempts.

*Please see later in the article for the Editors' Summary*

## Abstract 2

Karen Devries and colleagues conduct a systematic review of longitudinal studies to evaluate the direction of association between symptoms of depression and intimate partner violence.

*Please see later in the article for the Editors' Summary*

## Editors' Summary

**Background:**

Depression and suicide are responsible for a substantial proportion of the global disease burden. Depression—an overwhelming feeling of sadness and hopelessness that can last for months or years—affects more than 350 million people worldwide. It is the eleventh leading cause of global disability-adjusted life-years (a measure of overall disease burden), and it affects one in six people at some time during their lives. Globally, about a million people commit suicide every year, usually because they have depression or some other mental illness. Notably, in cross-sectional studies (investigations that look at a population at a single time point), experience of intimate partner violence (IPV, also called domestic violence) is strongly and consistently associated with both depressive disorders and suicide. IPV, like depression and suicide, is extremely common—in multi-country studies, 15%–71% of women report being physically assaulted at some time during their lifetime. IPV is defined as physical, sexual, or psychological harm by a current or former partner or spouse; men as well as women can be the victims of IPV.

**Why Was This Study Done?:**

It may seem obvious to assume that IPV is causally related to subsequent depression and suicidal behavior. However, cross-sectional studies provide no information about causality, and it is possible that depression and/or suicide attempts cause subsequent IPV or that there are common risk factors for IPV, depression, and suicide. For example, individuals with depressive symptoms may be more accepting of partners with characteristics that predispose them to use violence, or early life exposure to violence may predispose individuals to both depression and choosing violent partners. Here, as part of the Global Burden of Disease Study 2010, the researchers investigate the extent to which experience of IPV is associated with subsequent depression and suicide attempts and vice versa in both men and women by undertaking a systematic review and meta-analysis of longitudinal studies that have examined IPV, depression, and suicide attempts. A systematic review uses predefined criteria to identify all the research on a given topic, meta-analysis combines the results of several studies, and longitudinal studies track people over time to investigate associations between specific characteristics and outcomes.

**What Did the Researchers Do and Find?:**

The researchers identified 16 longitudinal studies involving a total of 36,163 participants that met their inclusion criteria. All the studies included women, but only four also included men. All the studies were undertaken in high- and middle-income countries. For women, 11 studies showed a statistically significant association (an association unlikely to have occurred by chance) between IPV and subsequent depressive symptoms. In a meta-analysis of six studies, experience of IPV nearly doubled the risk of women subsequently reporting depressive symptoms. In addition, there was evidence of an association in the reverse direction. In a meta-analysis of four studies, depressive symptoms nearly doubled the risk of women subsequently experiencing IPV. IPV was also associated with subsequent suicide attempts among women. For men, there was some evidence from two studies that IPV was associated with depressive symptoms but no evidence for an association between IPV and subsequent suicide attempt or between depressive symptoms and subsequent IPV.

**What Do These Findings Mean?:**

These findings suggest that women who are exposed to IPV are at increased risk of subsequent depression and that women who are depressed are more likely to be at risk of IPV. They also provide evidence of an association between IPV and subsequent suicide attempt for women. The study provides little evidence for similar relationships among men, but additional studies are needed to confirm this finding. Moreover, the accuracy of these findings is likely to be affected by several limitations of the study. For example, few of the included studies controlled for other factors that might have affected both exposure to IPV and depressive symptoms, and none of the studies considered the effect of emotional violence on depressive symptoms and suicide attempts. Nevertheless, these findings have two important implications. First, they suggest that preventing violence against women has the potential to reduce the global burden of disease related to depression and suicide. Second, they suggest that clinicians should pay attention to past experiences of violence and the risk of future violence when treating women who present with symptoms of depression.

**Additional Information:**

Please access these websites via the online version of this summary at http://dx.doi.org/10.1371/journal.pmed.1001439.

- This study is further discussed in a *PLOS Medicine* Perspective by Alexander Tsai
- The US National Institute of Mental Health provides information on all aspects of depression and of suicide and suicide prevention (in English and Spanish)
- The UK National Health Service Choices website provides detailed information about depression, including personal stories about depression, and information on suicide and its prevention; it has a webpage about domestic violence, which includes descriptions of personal experiences
- The World Health Organization provides information on depression, on the global burden of suicide and on suicide prevention, and on intimate partner violence (some information in several languages)
- MedlinePlus provides links to other resources about depression, suicide, and domestic violence (in English and Spanish)
- The charity Healthtalkonline has personal stories about depression and about dealing with suicide

## Background

Unipolar depressive disorders are the second leading cause of disease burden in women aged 15–44 y worldwide, and self-inflicted injuries are the seventh leading cause of disease burden [1]. Intimate partner violence (IPV) is also common, being reported by 15%–71% of women over their lifetime [2]. These conditions are linked—IPV experience is strongly and consistently associated with depression, including depressive symptoms and depressive disorders, and suicide in cross-sectional studies of women in both high- and lower-income settings [3]–[7]. There is less research on men, but cross-sectional studies also show that depressive symptoms are associated with IPV experience [8]. Several authors have speculated that the increased exposure to various forms of violence among women relative to men may help to explain the greater prevalence of depression, suicide attempts, and other common mental disorders in women versus men [9],[10].

While it is easy to assume that IPV is causally related to subsequent depression and suicidal behaviour, evidence suggests a more complex relationship. There are three modes of association, which are possible in any combination: (1) IPV exposure causes subsequent depression and suicide attempts, (2) depression and/or suicide attempts cause subsequent IPV, and (3) there are common risk factors for both IPV and depression and suicide attempts that explain the association between them.

Traumatic stress is the main mechanism by which IPV might cause subsequent depression and suicide attempts. Traumatic events can lead to stress, fear, and isolation, which in turn may lead to depression and suicidal behaviour [9]. A recent meta-analysis of three longitudinal studies provides support for this direction of association with depression, but this analysis pooled depressive disorders, depressive symptoms, and postpartum depression; included only a subset of known studies; and examined only one direction of association (that IPV is a risk factor for depression) [5]. To our knowledge there are no meta-analyses of the associations between IPV and suicide attempts.

Conversely, other studies suggest that women with severe mental health difficulties are more likely to experience violent victimisation [11],[12]. The same may hold for more minor forms of depression. Studies among US teenagers suggest that depression precedes first incidents of dating violence [13]. It is plausible that depressive symptoms may influence partner selection, such that young men and women are more accepting of partners with poor impulse control, conduct disorders, or other factors that predispose partners to use violence. Although it is clear that violence must precede completed suicides, most studies on violence and suicide actually measure suicide attempts, which could precede violent experiences.

Developmental and early life exposures to violence and other traumas may also play a role in predicting both violence and depression, for example, by contributing to the formation of insecure or disorganised attachment styles, which are associated with both increased IPV and depression risk [14],[15]. Although the mechanism remains unclear, women who have experienced childhood sexual abuse (CSA) also have an increased risk of subsequent experience of IPV [16]. Usually, longitudinal twin studies provide the best means of ruling out the confounding effect of early life factors, and two twin studies that have investigated exposure to general trauma suggest that traumatic events are causally associated with increased risk of major depressive disorder and suicide [9],[10],[17],[18]. However, to our knowledge no twin studies have examined the role of IPV victimisation specifically.

To assess the magnitude and direction of the relationship between IPV and depression and suicide attempts, we conducted a systematic review and meta-analysis of longitudinal studies examining the association of depression and suicide attempts with IPV experience in women and men. This study was conducted as part of the work of the Expert Working Group on Violence, for the Global Burden of Disease Study 2010 [19]. We aimed to (1) describe the characteristics of included studies, (2) report on magnitude and direction of association, and (3) document and explore potential sources of heterogeneity.

## Methods

### Searches

**Figure 1:**
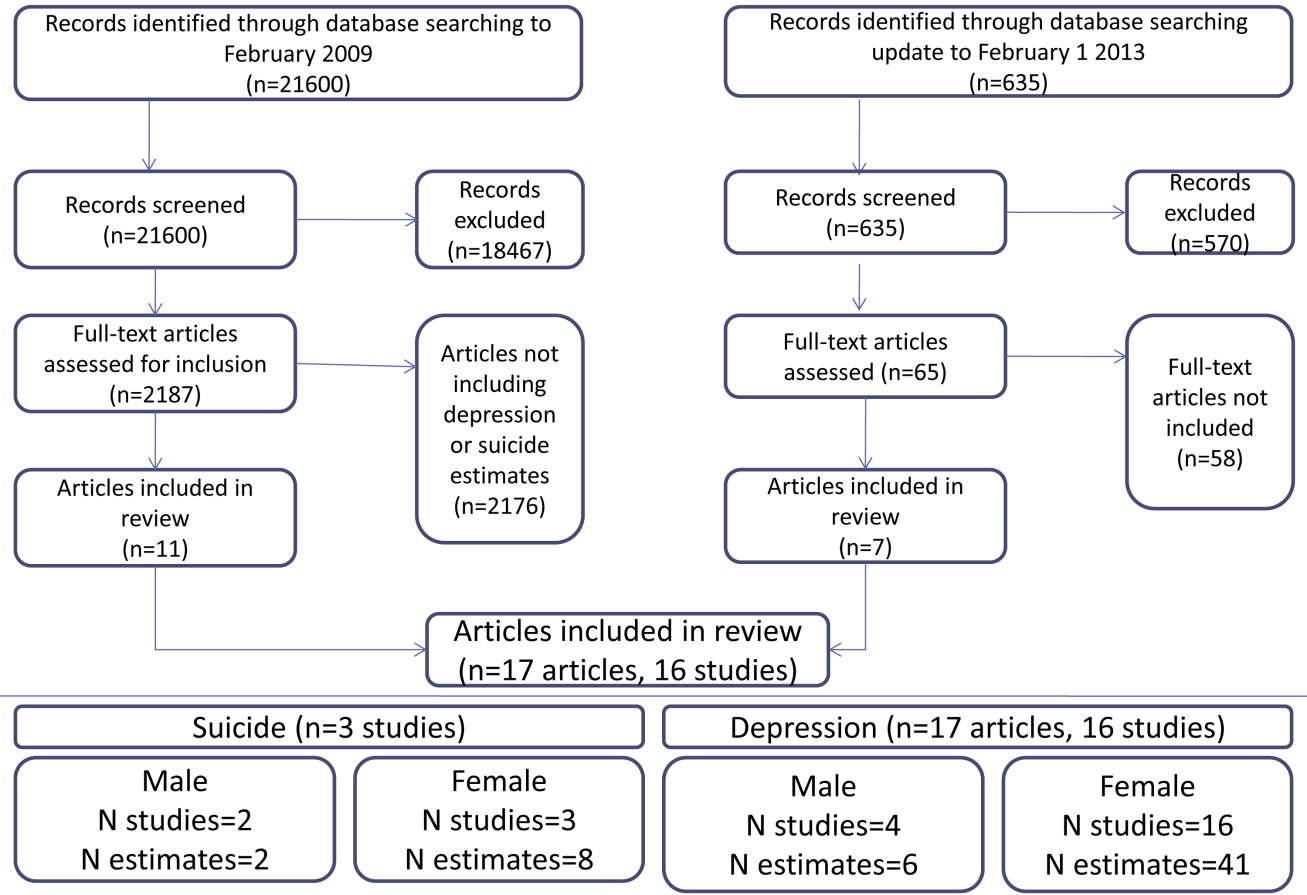
Flow of studies through the review.

We searched 20 different health and social science databases, including Medline, Embase, CINAHL (Cumulative Index to Nursing and Allied Health Literature), and region-specific databases from first record until February 1, 2009. This initial search was conducted as part of a larger set of systematic reviews, and included studies looking at health conditions in addition to depression and suicide. We updated the search in Medline to February 1, 2013, focusing on only depression and suicide studies. Strategies were designed in consultation with a librarian. Controlled vocabulary terms related to study design, violence, depression, and suicide were used for each database. The search and screening process is summarised in Figure 1. A list of databases and an example search strategy are provided in Text S2, and a PRIMSA checklist in Text S1.

### Inclusion Criteria

Longitudinal studies in any population of male and/or female participants were considered. Studies were deemed longitudinal if either the exposure or the outcome was measured at more than one time point. Papers reporting data from existing cohorts where both the exposure and outcome were assessed at the same time point were not included. All author definitions of IPV experience and all author definitions of depression (including symptoms and diagnoses) and measures of suicide attempts were eligible for inclusion. Papers reporting only on postpartum or antenatal depression were not included. Papers reporting only on suicidal thoughts or plans were not included.

### Screening and Data Extraction

For the original search, abstracts were screened by one reviewer; full text articles were appraised by JM, JC, GF, or LB and re-appraised by KMD. Data were extracted by one reviewer (JM, JC, GF, or LB) onto a standardised form, and checked by KMD. For the update, all steps were performed by KMD. Information about study population, exposure and outcome definitions, length of follow-up, effect estimates and uncertainty, analysis and control for confounding, and study quality were extracted.

### Quality Assessment

We appraised the quality of each effect estimate. We considered the definitions of the violence and depression/suicide measures and whether these were measured using valid, reliable instruments. We considered how the reference groups for each exposure were constructed (if they were truly unexposed or if there could potentially have been some misclassification). This is especially important for research examining the effects of IPV, as different forms of IPV (physical and sexual) are often only moderately correlated [2]. Studies measuring only one form of violence therefore potentially have a comparison group with exposure to the other form of violence.

We also considered control for potential confounders in key areas. First, because both IPV and depression commonly occur episodically over a period of time, events of either that are incident over the study period could be a continuation of previous violence/depression. Thus, we examined whether time one levels (at the beginning of the study period) of the outcome variable were adjusted for. Second, both IPV and depression/suicide attempts are associated with childhood adverse events, substance use, demographics, and other common risk factors that may explain the association between them. Because of the complexity of the potential causal pathways involved, we did not define a minimum set of confounders or common risk factors that should be adjusted for, but we aimed to consider results in light of which variables were included in analyses.

### Data Synthesis

Overall results on study characteristics and quality are summarised descriptively. Studies reported a range of different types of effect estimates (for example, relative risks, odds ratios [ORs], and correlation coefficients). They also varied on whether violence and depression outcomes were measured as binary or continuous variables, making it difficult to quantitatively summarise results. Where information was not reported, we calculated effect estimates and uncertainty as far as possible. Therefore, we present (1) results of all studies meeting the inclusion criteria in their original metrics in tabular format, and (2) where possible, pooled measures of effect using random effects meta-analysis. Heterogeneity was measured using Higgins *I* ^2^, with *p*<0.10 taken to indicate possible heterogeneity. For each meta-analysis, only one estimate per data source was included. The estimate least subject to bias according to the quality criteria above was selected. We had too few studies to quantitatively examine sources of heterogeneity.

### Ethics Statement

All data used in this review were already in the public domain; no ethical approval was required.

## Results

### Study Characteristics

Sixteen studies with 36,163 participants met the inclusion criteria. These were reported in 17 papers and contained 55 relevant effect estimates. Ten of these studies were from the US, two from Australia [20],[21], one from Sweden [22], one from India [23], one from Nicaragua [24], and one from South Africa [25]. Three studies from the US [13],[26],[27] included adolescents and focused on dating violence; all of the other studies focused on IPV in adults. Four studies sampled participants from secondary schools [13],[22],[26],[27], four studies were individual or household surveys of the general population [20],[21],[23],[28], one was conducted at a college [29], one was conducted among hospital employees [30], and three sampled from a variety of venues [25],[31],[32]. The three remaining studies recruited pregnant women, two from hospitals [33],[34] and one from households in the general population [24]. Details of study characteristics are described in Table S1.

The median follow-up time was 36 mo (interquartile range 12–60 mo) (range 2 mo [29] to 14 y [31]). Median attrition rate was 22.5% (interquartile range 17%–28.6%) (range 4.5% [31] to 57.1% [34]). Ten studies made use of two waves of data collection, two had three waves [23],[34], two had four waves [26],[30], one had five waves [33], and one had 14 [31]. The majority of studies included only female participants; the four studies that recruited from secondary schools also included males.

#### IPV measurement and potential misclassification

Nearly all (14 of 16) studies used measures of experience of specific acts of violence based in whole or in part on the Conflict Tactics Scale [35] or the World Health Organization instrument [24],[25]; one was based on the Abuse Assessment Screen [30] and one on the Severity of Violence Against Women Scales [33]. All of these instruments measure self-reported experience of specific acts of violence, for example, “Have you ever been slapped, punched, kicked, hit with an object”, and so on. Although measuring specific acts avoids misclassification associated with participants having different perceptions of whether what they have experienced constitutes “violence” or not [2], nearly half of all studies (six studies; seven papers) measured exposure to physical violence or sexual violence only [13],[28],[29],[31],[36],[37], leaving open the possibility of substantial misclassification of total violence exposure. Loxton et al. asked only if the respondent had “been in a violent relationship with a spouse” [20] and Jonsson et al. asked if participants “had ever been physically abused or had their life threatened” [22].

### Depression

Sixteen studies (reported in 17 papers—two papers used data from Add Health [13],[37]) provided 47 estimates of association between IPV and depressive symptoms or disorder. Forty-one estimates from 16 studies were for women, and six estimates from four studies were for male populations. Taking only the least biased estimate from each study gives 23 estimates from 16 studies. These 23 estimates are outlined in Table 1 and considered below; more detailed study information, including other effect estimates, is presented in Table S1.

**Table 1:** Summary of studies of depression and IPV, in women.

Study; Participants, Country	IPV Measure	Depression Measure	Effect Estimate (95% CI), *p*-Value^a^	Factors Adjusted For		
				Time One Depression	CSA	Trauma^b^
**IPV and incident depression**						
Ackard et al. [27] (Project Eat); 822 adolescents, US	Physical and/or sexual, CTS-like	Symptoms, “Kandel and Davies” scale, time period not specified	OR = 1.92 (1.22–3.00)	Yes (past year at baseline)	No	No
Chowdhary and Patel [23]; 1,563 adults, India	Physical^c^, CTS-like	CIS-R, past year	OR = 0.88 (0.26–3.00)	Yes	No	No
La Flair et al. [30]; 1,438 hospital employees, US	Physical and/or sexual, AAS	CES-D, past week	Beta = 0.65, *p* = 0.39	No	Yes	Yes
Levendosky et al. [33]; 150 adults, US	Physical and/or sexual, SVAWS	BDI, time period not specified	*r* = 0.24, *p*<0.05	No	No	No
Lindhorst and Oxford [31]; 229 young adults, US	Physical, CTS	BSI, past week	Beta = 0.23, *p*<0.001	Yes	No	No
Loxton et al. [20]; 11,648 adults, Australia	“Violent relationship”, not defined	CES-D, past year and/or “ever diagnosis”	OR = 2.51 (2.07–3.06)	Yes	No	No
Newcomb and Carmona [32]; 113 HIV+ and HIV− Latina women, US	Physical, CTS	CES-D, time period not specified	Path coefficient = 0.17, *p* = 0.05	Yes	Yes	No
Rich et al. [29]; 551 college students, US	Physical and verbal, CTS	BDI-II, time period not specified	Correlation = 0.11, *p* = 0.05; path analysis = “not significant”	Yes	Yes	Yes
Roberts et al. [37] (Add Health); 1,659 adolescents, US	Physical, CTS-like	CES-D, time period not specified	Beta = 0.18 (0.1–0.26)	Yes	No	No
Salazar et al. [24]; 276 adults, Nicaragua	Physical and/or sexual, CTS-like (WHO)	SRQ-20, time period not specified	OR = 2.01 (1.08–3.78), *p* = 0.0178	No	No	No
Suglia et al. [34]; 1,834 adults, US	Physical and/or sexual, CTS-like and general question	CIDI-SF, past year	Adjusted OR = 1.09 (0.6–1.9)	Yes	No	No
Taft and Watson [21]; 9,683 adults, Australia	Physical and/or sexual, CTS-like and general question	CES-D, time period not specified and “ever diagnosis”	OR = 2.12 (1.69–2.65)	Yes	No	No
Zlotnick et al. [28]; 3,104 adults, US	Physical, CTS-like	CES-D, past week	Beta = 6.96, *p* = 0.003	Yes	No	No
**Depression and incident IPV**						
Foshee et al. [26]; 1,965 adolescents, US	Physical^b^, CTS-like	Symptoms, “Kendler” scale, time period not specified	Hazard ratio = 1.35 (1.05–1.74)	Yes	Yes	Yes
Jonsson et al. [22]; 322 adults, Sweden	Physical, one general question	Any symptoms (BDI, CES-D-C, time period not specified) or diagnoses (DICA-R-A) or MINI	OR = 3.47 (1.11–10.84)	No	Yes	Yes
Nduna et al. [25]; 995 adults, South Africa	Physical and/or sexual, CTS-like (WHO)	CES-D, past week	Adjusted OR = 1.67 (1.18–2.36)	Yes	No	No
Lehrer et al. [13] (Add Health); 1,659 adolescents, US	Physical, CTS-like	CES-D, past week	Adjusted OR = 1.86 (1.05–3.29)	Yes	Yes	Yes
Levendosky et al. [33]; 150 adults, US	Physical and/or sexual, SVAWS	BDI, time period not specified	*r* = 0.23, *p*<0.05	No	No	No
Salazar et al. [24]; 370 adults, Nicaragua	Physical and/or sexual, CTS-like (WHO)	SRQ-20, time period not specified	OR = 2.42 (1.46–4.02), *p* = 0.002	Yes	No	No

a *p*-Value if no confidence interval reported.

b Other childhood trauma.

c More than one type of violence measured but only one estimate included here.

AAS, Abuse Assessment Screen; BDI, Beck Depression Inventory; BDI-II, Beck Depression Inventory–II; BSI, Brief Symptom Inventory; CES-D-C, Center for Epidemiologic Studies–Depression–Children; CI, confidence interval; CIDI-SF, Composite International Diagnostic Interview–Short Form; CIS-R, Clinical Interview Schedule–Revised; CTS, Conflict Tactics Scale; DICA-R-A, Diagnostic Interview for Children and Adolescents–Revised-Adolescents; MINI, Mini-International Neuropsychiatric Interview; SVAWS, Severity of Violence Against Women Scales; WHO, World Health Organization.

#### Depression measurement

Of the 16 studies included, eight measured depressive symptoms over a defined time period (five were over the 1 wk prior to the survey, three were over the past year, and the remainder did not specify). Seven studies used the Center for Epidemiologic Studies Depression Scale (CES-D) [13],[20],[21],[25],[28],[30],[36], two used the Beck Depression Inventory [29],[33], one used the Self-Report Questionnaire–20 (SRQ-20) [24], one used the Brief Symptom Inventory [31], one used the Composite International Diagnostic Interview–Short Form [34], one used a scale from K. S. Kendler [26], and one used a scale from D. B. Kandel and M. Davies [27]. The one study that measured incident depressive disorders [23] used the Clinical Interview Schedule–Revised. Jonsson et al. used the CES-D and Beck Depression Inventory but also the Diagnostic Interview for Children and Adolescents–Revised–Adolescents and the Mini-International Neuropsychiatric Interview. All measures were combined for analysis [22].

#### Common risk factors/confounding

Of the estimates for women, presented in Table 1, most were adjusted for time one measures of the outcome, but five estimates were unadjusted. Chowdhary and Patel [23] excluded lifetime suicide and depressive disorder diagnosis at baseline from analyses; however, this likely resulted in the exclusion of many cases of violence that preceded suicide attempts or depressive symptoms or disorder at baseline—the resulting cases of violence being few and not representative of women experiencing IPV. Nearly all studies (14 of 16) also controlled for demographic factors, but in general, other confounders were not comprehensively controlled. Often the estimates included in the meta-analyses, only two controlled for CSA and/or other early life experiences [13],[22]. None controlled for alcohol use. Of the seven studies not included in the meta-analyses (those with continuous measures of depression), 5/7 controlled for demographic factors [28]–[31],[37], but only 2/7 for CSA [29],[30], one for early life factors [30], and one for early risk behaviour [37]. Despite these differences in variables controlled for across analyses, there were no discernible differences in effect estimates: regardless of which confounders were adjusted for, all studies found similar directions and varying magnitudes of association. For men (Table 2), the picture was similar: most studies adjusted for time one levels of the outcome, but other key confounders were not adjusted for.

**Table 2:** Summary of studies of depression and IPV, in men.

Study; Participants, Country	IPV Measure	Depression Measure	Effect Estimate (95% CI), *p*-Value^a^	Factors Adjusted For		
				Time One Depression	CSA	Trauma^b^
**IPV and incident depression**						
Ackard et al. [27] (Project Eat); 694 adolescents, US	Physical and/or exual, CTS like	Symptoms, “Kandel and Davies” scale, time period not specified	OR = 1.92 (1.22–3.00)	Yes	No	No
Roberts et al. [37] (Add Health); 2,237 adolescents, US	Physical, CTS-like	CES-D, time period not specified	Beta = 0.18 (0.1–0.26)	Yes	No	No
**Depression and incident IPV**						
Foshee et al. [26]; 638 adolescents, US	Physical^c^, CTS-like	Symptoms, “Kendler” scale, time period not specified	Hazard ratio = 1.1 (0.9–1.36)	Yes	No	No
Jonsson et al. [22]; 84 adults, Sweden	Physical, one general question	Any symptoms (BDI, CES-D-C, time period not specified) or diagnoses (DICA-R-A) or MINI	2.8% of those with versus 0% of those without depression at wave 1 reported IPV at wave 2	No	No	No

a *p*-Value if no confidence nterval reported.

b Other childhood trauma.

c More than one type of violence measured but only one estimate included here.

BDI, Beck Depression Inventory; CES-D-C, Center for Epidemiologic Studies–Depression–Children; CI, confidence interval; CTS, Conflict Tactics Scale; DICA-R-A, Diagnostic Interview for Children and Adolescents–Revised–Adolescents; MINI, Mini-International Neuropsychiatric Interview.

#### Effect estimates for depressive disorder and symptoms in women

Of the 16 studies looking at depressive symptoms or disorder and IPV in women, 13 provided estimates of IPV and incident depressive symptoms or disorder and six provided estimates of depressive symptoms and incident IPV (Table 1). Twelve of 13 estimates showed a positive direction of association between experience of IPV and incident depressive symptoms, with 11 reaching statistical significance. All six estimates looking at depressive symptoms and incident IPV also showed positive associations, which were statistically significant.

**Figure 2:**
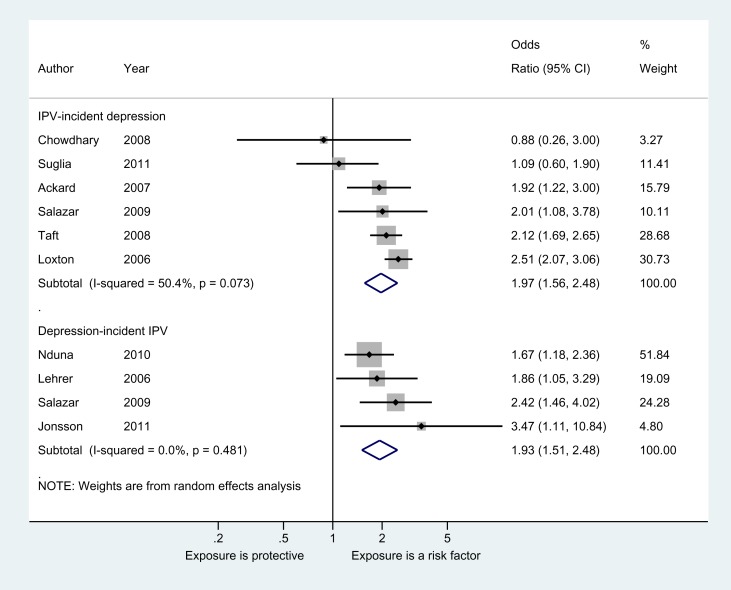
Meta-analyses of the association between IPV and depressive symptoms or disorder in women.

We were able to include all estimates reporting binary violence measures and binary depressive symptoms or disorder measures in meta-analyses (Figure 2). For IPV and incident depressive symptoms or disorder, the pooled OR from six estimates was 1.97 (95% CI 1.56–2.48). This was heterogeneous (*I* ^2^ = 50.4%, *p* = 0.073), although almost all studies had a positive direction of effect. Removing the outlier (Chowdhary and Patel [23]) did not improve heterogeneity estimates. Four estimates were included in the meta-analysis of the relationship between depressive symptoms and incident IPV, resulting in a pooled OR of 1.93 (95% CI 1.51–2.48, *I* ^2^ = 0%, *p* = 0.481).

#### Effect estimates for depressive symptoms in men

For men (Table 2), two studies [27],[37] examined experience of IPV and incident depressive symptoms, and both studies showed a significant association in a positive direction. Foshee et al. examined depressive symptoms and time to onset of physical and sexual victimisation, as well as “chronic victimisation”, and found non-significant relationships in a positive direction (bivariate model) [26]. Jonsson et al. found that 2.5% of adult men reporting depressive symptoms as adolescents also reported adult experiences of IPV, versus 0% of adult men who did not report depressive symptoms in adolescence [22].

### Suicide Attempts

Three studies investigating suicide attempts met our inclusion criteria [23],[27],[37]. These studies reported eight estimates of association of experience of IPV with incident suicidal attempts. Six were for female populations, and two were for male populations. Ackard et al. and Roberts et al. both sampled US adolescents and the IPV measured was dating violence (for both male and female adolescents) [27],[37]. Chowdhary and Patel sampled a cohort of adults from Goa, India, comprising adult women only [23]. No studies examined suicide attempts and incident IPV.

#### Suicide measures

All studies modelled lifetime suicide attempts as a binary variable, and assessed attempts with a single question. No studies had completed suicides as an outcome.

#### Common risk factors/confounding

Ackard et al. [27] and Roberts et al. [37] controlled for time one suicide attempts; Chowdhary and Patel [23] excluded participants with lifetime suicide attempts at baseline. None controlled for early life factors, including experience of CSA.

#### Effect estimates for suicide attempts in women

Chowdhary and Patel [23], Ackard et al. [27], and Roberts et al. [37] examined violence and incident suicide attempts: all three studies showed positive relationships, of which two were statistically significant and one was of borderline significance (Table 3).

**Table 3:** Summary of studies of suicide and IPV, in women.

Study; Participants, Country	IPV Measure	Suicide Attempts Measure	Effect Estimate (95% CI), *p*-Value^a^	Factors Adjusted For		
				Time One Suicide Attempts	CSA	Trauma^b^
Ackard et al. [27] (Project Eat); 822 adolescents, US	Physical and/or sexual, CTS-like	Single question, ever attempted suicide	OR = 3.2 (0.97–103.59)	Yes	No	No
Chowdhary and Patel [23]; 1,563 adults, India	Physical^c^, CTS-like	Single question, ever attempted suicide	OR = 7.97 (1.75–36.37)	Yes	No	No
Roberts et al. [37] (Add Health); 1,659 adolescents, US	Physical, CTS-like	Single question, ever attempted suicide	Beta = 0.12 (0.02–0.22)	Yes	No	No

All studies measured IPV and incident suicide attempts.

a *p*-Value if no confidence interval reported.

b Other childhood trauma.

c More than one type of violence measured but only one estimate included here.

CI, confidence interval; CTS, Conflict Tactics Scale.

#### Effect estimates for suicide attempts in men

Two studies examined violence and incident suicide attempts [27],[37]: both found non-significant relationships, one in a positive direction and the other with exactly no association (Table 4). Both of these studies included adolescent or young adult US men; both also controlled for time one suicide attempts.

**Table 4:** Summary of studies of suicide and IPV, in men.

Study; Participants, Country	IPV Measure	Suicide Attempts Measure	Effect Estimate (95% CI), *p*-Value^a^	Factors Adjusted For		
				Time One Suicide Attempts	CSA	Trauma^b^
Ackard et al. [27] (Project Eat); 694 adolescents, US	Physical and/or sexual, CTS-like	Single question, ever attempted suicide	OR = 7.55 (0.81 to 70.31)	Yes	No	No
Roberts et al. [37] (Add Health); 2,237 adolescents, US	Physical, CTS-like	Single question, ever attempted suicide	Beta = 0 (−0.6 to 0.6)	Yes	No	No

All studies measured IPV and incident suicide attempts.

a *p*-Value if no confidence interval reported.

b Other childhood trauma.

CI, confidence interval; CTS, Conflict Tactics Scale.

## Discussion

### Summary of Main Findings

Our review provides evidence that experience of IPV increases the odds of incident depressive symptoms and of suicide attempts among women. We also found evidence that depressive symptoms can increase the odds of incident IPV in women. However, our ability to draw firm conclusions is limited by the quality of the available studies, in particular the lack of adjustment for common risk factors. Relatively few studies included men, but these studies suggested a relationship between IPV and incident depressive symptoms. For men, there was no clear evidence of an association between IPV and incident suicide attempts, or between depressive symptoms and incident IPV.

### Limitations of This Review

Our review employed extensive searches of global literature in multiple languages. Despite this, our review has some limitations. Because of the large volume of search results returned, we were unable to employ double screening of abstracts, and for our update, double data extraction. We also did not contact authors for additional information. The different scales of measurement (binary or continuous) employed across various studies meant that we were unable to combine all measures of effect, which limited the number of studies in our meta-analyses. However, studies that we could not include in meta-analyses showed a positive direction of effect consistent with that of the studies included in the meta-analyses. Too few studies met the inclusion criteria to meaningfully assess publication bias.

### Sources of Bias and Limitations of Included Studies

The main limitation of included studies relates to lack of comprehensive control of potential confounders. Both IPV and depression can be conceptualised as chronic episodic conditions, and most studies controlled for time one levels of the outcome variable or excluded baseline cases in their analyses. However, alcohol use and childhood adversity, including early experiences of violence and trauma, were generally not controlled for, making it difficult to rule out these other factors as contributors to the causation of outcomes. We did find that studies generally showed a positive direction of association regardless of which potentially confounding variables were adjusted for, and there was also no clear pattern of differing magnitude of association, indicating that the relationships between IPV and depressive symptoms and suicide are not likely to be entirely accounted for by shared risk factors.

Almost all included studies on depression measured depressive symptoms rather than major depressive disorder, dysthymia, or other depressive disorders using Diagnostic and Statistical Manual of Mental Disorders or International Classification of Diseases diagnostic criteria. Only around one-third to one-half of people who score above recommended CES-D cutoffs are diagnosed with major depressive disorder [38],[39]. The relationship between violence experience and depressive disorders may differ from the relationship between violence experience and depressive symptoms. Major depressive disorder has a substantial heritability [40] and has been shown to be more heritable than less severe forms of depression [41]; situational causes such as violence may therefore play a more important etiological role in the less severe forms of depression. Conversely, experiences of violence may predict more severe depression and thus have a stronger association with depressive disorders than with depressive symptoms. When examining violence in relation to depression, including subthreshold depressive symptoms and depressive disorders could either dilute or inflate effect estimates.

Most studies were from high-income countries, and four were of adolescents or college students. In high-income contexts, in these samples, relationships will be primarily in dating relationships. In dating relationships where there is no cohabitation, there may be a lower likelihood of chronic exposure to violence within the intimate partnership [42],[43], which may lessen any subsequent mental health impact. Other studies of the features of intimate partner abuse have shown that fear, entrapment, and feelings of inability to escape from violent situations specifically contribute to increased adverse mental health outcomes [42]—these relationship features are likely to be less pronounced in dating relationships, which could mean that effects are underestimated in studies including only adolescents.

Emotional violence, which we did not include here, may also be an important predictor of adverse mental health outcomes [44]. The epidemiological study of emotional IPV is in its infancy, but at least one study that has modelled combined measures of physical, sexual, and emotional IPV has shown a relationship between these forms of abuse and incident suicide attempts in Indian women [45].

Most studies also measured exposure only to physical violence, or modelled exposure to physical violence and sexual violence separately. Most studies constructed reference categories as binary opposites, meaning that some participants in the reference group may have been exposed to other forms of violence by intimate partners that were not measured or modelled. This approach may bias the effect estimates towards the null, and underestimate the magnitude of the association between violence experience and depression outcomes. Several studies also included only women who were in relationships for all time points of data collection. The prevalence of IPV is usually higher in women who no longer have a partner versus women currently in a partnership (for example, [46]). Not including these women may bias associations towards the null. Similarly, it is conceivable that women who are no longer in a partnership may have higher or lower odds of depression/suicide attempts. If they are not surveyed in subsequent waves, associations may be biased in different directions.

### Is the Relationship between IPV, Depression, and Suicide Causal?

Cross-sectional evidence suggests that lifetime experience of IPV is consistently associated with both SRQ-20 score (representing probable cases of depression and/or anxiety) [4] and suicide attempts among women in a range of low- and middle-income countries [3]. Several studies have shown a dose–response relationship, where IPV is associated with increased frequency of depressive episodes [20], and other studies have shown that depression is more strongly predictive of incident severe IPV than it is of less severe IPV [13]. Twin studies provide evidence for a plausible causal mechanism, that exposure to traumatic events, including sexual assault and violence, can cause increased risk of depression, ruling out early life confounders [10],[18].

Our review presents evidence for a temporal relationship between IPV experience and depressive symptoms, but also shows that women with existing depressive symptoms are more likely to subsequently experience IPV. Our finding is consistent with other longitudinal studies that have considered combined measures of IPV perpetration and experience, which found that women with depression were more likely to be in an abusive relationship, but also that being in an abusive relationship predicted incident major depressive disorder [47]. In summary, it seems that the relationship between IPV and depression is bidirectional, with women who are exposed to IPV being at increased risk of depression symptoms, and women who report depressive symptoms being more likely to subsequently experience IPV. For young men, we found no clear evidence of a relationship between IPV, depressive symptoms, and suicide, but very few studies included men. Further studies that include male participants are needed to clearly establish whether or not there is an association.

### Implications

The different forms of depression—major depressive disorder, dysthymia, and mild depression—as well as suicidal behaviour, are some of the largest causes of disease burden in women globally. Our findings suggest that interventions to prevent violence need to be explored for their efficacy in reducing different forms of depression. Similarly, for women already receiving mental health treatments or presenting with symptoms of depression, attention must be paid to experiences of violence and risk of future violence. Because IPV often occurs as a pattern of ongoing events [43], treatment strategies that fail to address womens' experience of violence may do harm. For example, if violence is not suspected as a potential causative factor, patients who have attempted suicide may be encouraged to return to partners/relatives, which could increase the risk of further violence and eventual suicide [48]. Anti-depressant medication may also interfere with women's ability to make decisions about how to respond to violence [49].

Further research is needed to explore why having depressive symptoms can lead to incident violence—it may be that young women with depressive symptoms are predisposed to choose partners who use violence. Depression can also lead to maladaptive coping with stress, cognitive distortions about risk, and loss of self-efficacy. Young people who have experienced early traumatic events, including violence in their families, are at higher risk for poor mental health as adolescents [50]. Longitudinal studies where both violence exposure and depression are measured at multiple time points are needed to more clearly elucidate causal mechanisms. It is clear that addressing the burden of untreated mental disorders in a population could have substantial effects on the prevalence of violence.

### Conclusion

Interventions to prevent violence should be explored for their efficacy in reducing the burden of depressive symptoms and disorders as well as suicide attempts in women. Women who have experienced violence may benefit from tailored interventions that address the changes that come with prolonged exposure to trauma in order to prevent future depression and suicidal behaviour.

## Supporting Information

Text S1 **PRISMA checklist.**
(DOCX)

Text S2 **List of databases searched and example search strategy.**
(DOCX)

Table S1 **Details of longitudinal studies included in the review.**
(DOCX)

## Abbreviations

CES-D: Center for Epidemiologic Studies Depression Scale
CSA: childhood sexual abuse
IPV: intimate partner violence
OR: odds ratio
SRQ-20: Self-Report Questionnaire–20
