# Supplementary material for: Intimate Partner Violence and Incident Depressive Symptoms and Suicide Attempts: A Systematic Review of Longitudinal Studies
Source: PLoS Med. 2013 May 7;10(5):e1001439. doi: 10.1371/journal.pmed.1001439 (PMC3646718; doi:10.1371/journal.pmed.1001439)
Supplement: Text S2 — List of databases searched and example search strategy. (DOCX) [file pmed.1001439.s002.docx]

**Text S2. List of databases searched and example search strategy.**

African Healthline, Applied Social Sciences Index and Abstracts, British Nursing Index, Cochrane Library, Cumulative Index to Nursing and Allied Health Literature, Embase, Health Management Information Consortium, Index Medicus for the Eastern Mediterranean Region, Index Medicus of the South-East Asian Region, International Bibliography of Social Sciences, LILACS, MedCarib, Medline, Midwives Information and Resource Service, NHS Library for Health Specialist Libraries, Popline, PsychINFO, Science Direct, Web of Science, Western Pacific Region Index Medicus, and Wiley InterScience.

Example search strategy from Medline (via OVID):

1. Animals/

2. Humans/

3. 1 not (1 and 2)

4. comment.pt.

5. letter.pt.

6. editorial.pt.

7. or/3-6

8. domestic violence/ or partner violence/ or spouse abuse/ or spouse violence/ or domestic abuse/ or partner abuse.mp.

9. *battered women/

10. (intimate adj4 partner adj4 violence).tw.

11. domestic abuse.tw.

12. spou$ abuse.tw.

13. ((partner or relationship or wom$n or domestic or spous*) adj4 (abus* or violen* or victimi* or batter*)).mp.

14. ((partner or relationship) adj4 (sexual abus* or sexual victimi* or sexual violence or rape)).mp.

15. (intimate adj4 partner adj4 abuse).tw.

16. (intimate adj4 partner adj4 victimi*).tw.

17. dating violence.tw.

18. or/8-17

19. Depression/

20. (unipolar adj4 depres$).tw.

21. suicid$.tw.

22. Suicide/ or Suicide, Attempted/

23. (emotion$ adj4 distress).tw.

24. parasuic$.tw.

25. or/19-24

26. (child* adj4 sexual adj4 abuse).tw.

27. (((sexual or dating) adj4 violence) or date rape).mp.

28. (dating adj4 violence).tw.

29. Child Abuse, Sexual/

30. or/26-29

31. ((18 or 30) and 25) not 7
