# Supplementary material for: Intimate Partner Violence and Incident Depressive Symptoms and Suicide Attempts: A Systematic Review of Longitudinal Studies
Source: PLoS Med. 2013 May 7;10(5):e1001439. doi: 10.1371/journal.pmed.1001439 (PMC3646718; doi:10.1371/journal.pmed.1001439)
Supplement: Table S1 — Details of longitudinal studies included in the review. (DOCX) [file pmed.1001439.s003.docx]

Table S1. Details of longitudinal studies included in the review

| Data source | Measure IPV | Measure Depression/  Suicide attempts | Association | Covariates | Comments |
| --- | --- | --- | --- | --- | --- |
| Ackard [[27](#_ENREF_27)]  USA, Project Eat.  694 male and 822 female middle and high school students  Wave 1=1999  Wave 2=2004  Participation rate=81.5%  Attrition rate=18.2% | Physical and/or sexual IPV, before wave 1only.  Physical: “Have you ever been hit, shoved, held down or had some other physical force used against you by someone you were dating?”  Sexual: “In a dating relationship, have you ever been forced to touch your date sexually or have they forced some type of sexual behavior on you?” | Suicide “Have you ever tried to kill yourself?”  Only in past year included in analysis.  Depression:  Scale from Kandel and Davies, included fatigue;  sleep disturbance; dysthymic mood; hopelessness; feeling  tense/nervous; and worry; time period not specified. | Suicide attempts  Female:  aOR=3.20 (0.97-10.59), p=0.057  Male:  aOR=7.55 (0.81-70.31), p=0.076  **Depressive symptoms:**  **Female**:  **aOR=1.92 (1.22-3.00)**, p=0.005  Male: aOR=2.08 (0.80-5.41), p=0.132 | Wave 1 suicide attempts  Wave 1 depressive symptoms | Adolescent who reported IPV in past year at Wave 2 were excluded—all IPV preceded suicide attempts |
| Chowdhary [[23](#_ENREF_23)]  India, Goa cohort. 1563 married women aged 18-50 recruited from population register of Aldona Primary Care Centre.  Study period 2001-2004  Wave 1=baseline  Wave 2=+6 months  Wave 3=+1 year  Participation rate=83.1%  Attrition rate=10.0% | Physical, sexual IPV, lifetime and past 3 months exposure at baseline.  Physical: “Has your husband/partner ever hit you (Examples to probe: slap, hit, kick, pinch, pull your hair, etc)?”  Sexual: “Has your husband/partner ever forced you to have sex with him i.e. made you have sex against your wishes?” | Depression:  CIS-R (Revised Clinical Interview Schedule), using ICD-10 criteria to diagnose depressive disorder; incident, past year.  Attempted suicide, incident, past year | **Depressive disorder**:  Physical violence, lifetime:  aOR=0.88 (0.26-3.00)  past 3 months: aOR=0.50(0.07-3.79)  Sexual violence, lifetime:  aOR=0.88 (0.12-6.72)  Past 3 months: aOR=1.09 (0.14-8.36)  **Attempted suicide**:  Physical violence, lifetime:  aOR=7.97 **(1.75-36.37)**  Past 3 months: aOR=6.56 **(1.19-35.99)**  Sexual violence, lifetime,  aOR=10.91 **(2.01-59.30)**  Past 3 months: aOR=12.91 **(2.32-71.80)** | Age, literacy, household per capita income | 12 month incident cases depression n=33, total n=1466; attempted suicide n=9, total n=1537.  Paper also reports violence prevalence.  Outcome is incident cases; appears to have excluded people with history of depression, suicide at baseline from analysis. |
| Foshee [[26](#_ENREF_26)]  USA, 14 public schools in a rural North Carolina county, 653 female, 638 male followed from grade 8 or 9 to 12.  Wave1=1994  Wave 2-5=yearly assessments until 12^th^ grade (5 for Grade 8 cohort and 4 for Grade 9 cohort).  Participation rate=81%  Attrition rate=23% | Physical, sexual dating violence, modelled as continuous variables, lifetime.  Physical: ‘‘tried to choke me, burned me, hit me with a fist, hit me with something hard besides a fist, beat me up, and assaulted me with a knife or gun”.  Sexual: ‘‘forced me to have sex, forced me to do other sexual things that I did not want to do’’. | Depression:  Kendler’s 4 point scale.  “During the past 6 months, how much have you been bothered  or troubled by: (e.g., feeling unhappy, sad, or depressed,  feeling hopeless about the future)?” | Women:  **Depression-onset** physical victimisation: HR=1.10 (0.87–1.37)  Chronic physical victimisation  b=0.11 p=0.26  **Depression-onset** sexual victimisation **HR=1.39 (1.14– 1.69)**  Chronic sexual victmisation  **b= 0.31 p=0.0003**  **Depression-onset** physical victimisation=NS  Depression-chronic physical victimisation=NS  **Depression- onset** sexual victimisation **HR=1.35 ( 1.05– 1.74)**  **Depression- chronic** sexual victimisation **b=0.29 p=0.01**  Men:  Depression-onset physical victimisation HR=1.10 (0.90– 1.36)  Chronic victimisation  b= 0.12 p=0.20  Depression-onset physical victimisation=NS  Depression-chronic physical victimisation=NS | Unadjusted  Unadjusted  Unadjusted  Unadjusted  17 predictor variables  17 predictor variables  17 predictor variables  17 predictor variables  Unadjusted  Unadjusted  17 predictor variables  17 predictor variables | Have not reported non-significant multivariate results, but some bivariate non-significant results are reported. |
| Jonsson [[22](#_ENREF_22)]  Sweden, Uppsala cohort. 322 female and 84 males at follow-up, recruited from high school  Wave 1=1991-1993  Wave 2= 2006-2008  Participation rate=90.5%  Attrition rate=34.0% | Physical IPV, at wave 2.  Physical: “Have you ever been physically abused/threatened to your life?” “By whom?” “When?” | Depression, wave 1: Positive answer to screening BDI, CES-D-C at baseline or DSM III diagnosis from DICA-R-A.  Recurrent depression, wave 2. Defined as at least 2 episodes or >6 months in duration since age 19 years, using MINI | **Depressive symptoms and/or diagnosis:**  **Female:**  **aOR=3.47 (1.11-10.84)**  Depressive symptoms and/or diagnosis:  Male:  2.8% of those with vs. 0% of those without adolescent depression at wave 1 report IPV at wave 2. Fisher’s exact test, not significant. | Parental unemployment/economic hardships, conflicts with parents, physical/sexual abuse at home, child and adolescent disruptive disorder | Have combined depressive symptoms and diagnoses in analysis. Have not controlled for time 1 intimate relationship violence. May include never-partnered women in denominator. Additional sub-analyses conducted among depressed subsample with and without comorbid disruptive disorders for recurrent depression and IPV. |
|  |  |  |  |  |  |
| La Flair [[30](#_ENREF_30)]  USA, Maryland, 1438 female health workers from 3 hospitals; sample from a case-control study; cases were those who reported workplace violence; controls reported no violence.  Wave 1=2007  4 waves, each 6 months apart  Wave 4=2009  Participation rate=52%  Attrition rate=30% | Physical and/or sexual violence, Abuse Assessment Screen, assessed at Wave 1, measuring violence prior to Wave 1. | Depression: CES-D, 10 item version, past week (assumed to be at each wave) | IPV-depression  β=0.65 se=0.39, p>0.05  Time* IPV  β=0.08 se=0.03, p<0.05 | Time from baseline measurement, age, marital status, childhood physical, sexual abuse, IPV, time*IPV, child witness to IPV | IPV is related to change in CES-D over time  Only main effect is included in meta-analysis. |
| Lehrer[[13](#_ENREF_13)]  USA, National Longitudinal Study of Adolescent Health, 1659 young women in an opposite sex partnership at Wave 2 data collection.  Wave 1=1995  Wave 2=1996  Wave 3=2002  Participation rate=not reported  Attrition rate=18% | Physical violence, any past year experience, assessed at Waves 2 and 3.  Physical: “how often in the past year their current partner had threatened them with violence, pushed or shoved them, or thrown something at them that could hurt.” Classified as mild or moderate/severe. | Depression: modified 19-item CES-D, past-week; assessed at Wave 2, modelled as binary and continuous variable. | Depression- incident mild IPV:  aOR=1.49 (0.91-2.45)  Depression- incident moderate/severe IPV:  **aOR= 1.86 (1.05-3.29), p<0.05** | Age, race, wave 1 parental education, child physical abuse before 6^th^ grade, child sexual abuse before 6^th^ grade, dating violence/forced sex between waves 1 and 2. | Excluded 3 women who reported partner beat them previously but not in past year, so all IPV is truly incident. |
| Levendosky[[33](#_ENREF_33)]  USA.  150 women recruited at pregnancy  Wave 1=year not specified (pregnancy)  Waves 2-5=+1 year  Participation rate=not reported  Attrition rate=27.2% | Physical and/or sexual IPV and threats, at all 5 waves. SWAWS (Severity of Violence Against Women Scales), 46 items. | Depressive symptoms: BDI, at all 5 waves, time period not specified. | Depression Wave 1-IPV Wave 4, **r=0.23, p<0.05**  IPV Wave 1-Depression wave 5, **r=0.24, p<0.05** | Unadjusted. | Correlations between IPV and Depression for all waves provided; longest reported follow-up times extracted here. |
| Lindhorst [[31](#_ENREF_31),[51](#_ENREF_51)]  USA, Seattle, 229 adolescent mothers  Wave 1 1988-1990; 14 waves 6 months to 1 year apart (end year not specified)  Participation rate=not reported  Attrition rate=4.5%  Also reported in Gavin [[51](#_ENREF_51)], but only IPV and depressive symptoms | Physical violence, CTS, retrospective measure at age 18; measured prospectively at each wave after age 18; modelled as continuous variable.  Physical: “if the father of the respondent’s baby, her husband, or any boyfriend or sexual partner had since pregnancy: threatened to hit or throw something at her, threw something at her, pushed/ grabbed/ shoved/ or slapped her, hit her with a fist / object/ kicked/ or bit her, beat her up, threatened her with a knife or gun, or used a knife or fired a gun at her.” | Depression: BSI, past week, at each wave; CES-D at one time point—was highly correlated with BSI.  Depression modeled as a t-score at age of 28.4 years. | Adolescent IPV-depression:  β=0.02, p>0.05  Cumulative adult IPV-depression:  **β=0.23, p<0.001**  Concurrent IPV-depression:  **β=0.17, p<0.01** | Vulnerability to depressive symptoms (early reports of depression), other forms of IPV, cumulative welfare use, concurrent welfare use. | IPV prevalence. 67.2% of adolescent  mothers reported at least one episode of victimization. From pregnancy to age 18.  On average, respondents reported decreasing exposure  to IPV as time passed, with 15.0% reporting IPV at  the final time point.  Cumulative adult IPV included in summary analysis. |
| Loxton [[20](#_ENREF_20)]  Australia, Australian Longitudinal Study of Women’s Health, 11648 adult women  Wave 1=1996  Wave 2=1998  Participation rate=not reported Attrition=24% | Type of violence not defined; lifetime exposure.  Violence: “have you ever been in a violent relationship with a partner and/or spouse?” | Depression: Self-reports of ever having been diagnosed with depression by doctor or nurse, lifetime.  CES-D, past year | Ever being diagnosed with depression:  **aOR= 1.93 (1.70- 2.19)**  Frequency of past-year symptoms:  Rarely vs. never  **aOR=1.49 (1.29-1.73)**  Sometimes vs. never:  **aOR=1.99 (1.73- 2.29)**  Often vs. never:  **aOR=2.51 (2.07, 3.06)** | Marital status, income management, area of residence. | 254 women who experienced violence between Waves 1 and 2 excluded; 84 women with missing violence data excluded. All violence exposure occurred before Wave 2.  Have included ‘often’ in meta-analysis (closest to actual past year diagnosis) |
| Nduna[[25](#_ENREF_25)]  South Africa, Stepping Stones cohort.  995 young adults.  Wave 1=2002  Wave 2=2003  Participation rate=not reported  Attrition rate=22% | Physical and/or sexual IPV, adapted WHO instrument. Modelled as binary variable, experience of 2 or more acts of violence vs 1 or none.  Measured lifetime exposure at baseline and past year exposure at follow-up. | CES-D, past week, at baseline and follow-up | IPV at wave 2:  Female:  aOR=1.67 (1.18-2.36), p=0.003 | Baseline IPV, study design characteristics | IPV is incident. |
| Newcomb [[32](#_ENREF_32)]  USA, 79 HIV+ and 34 HIV- Latinas  Wave 1=not specified  Wave 2=+1 year  Participation rate=not reported  Attrition rate=36/149=24.1% | Physical violence, some items from CTS, assessed at Wave 1.  Physical:“threw, smashed, hit, kicked something; slapped/physically attacked/hurt the other one; and threatened with or used a knife or gun.” | Depression: CES-D, time period of recall not specified, assessed at waves 1 and 2. | IPV-Wave 2 depression: **path coefficient=0.17, p<0.05** | Wave 1 depression, adult sexual abuse, acculturation, illicit drug use, alcohol problems, depression, HIV status, age, education, relationship status | Path Model; controls for baseline depression. |
| Rich [[29](#_ENREF_29)]  USA, 551 college women from Midwest  Wave 1=not specified  Wave 2=+2 months  Participation rate=not reported  Attrition rate=5% | Physical and verbal dating violence: CTS | Depression: Beck depression inventory, assessed at Wave 1 and Wave 2  DSM-IV diagnostic criteria (but not used in analysis). | Correlation between adolescent dating violence and BDI-time 2**=0.11, p=0.05**  Correlation between adolescent dating violence before time 1 to time 1 and 2 depression, not significant | All variables in path model: child sexual abuse, mother verbal abuse, father verbal abuse, mother physical abuse, father physical abuse, adolescent dating violence, adolescent sexual victimization, depression time 1, dating violence follow-up, sexual victimization follow-up, depression time 2. |  |
| Robert [[37](#_ENREF_37)]  USA, National Longitudinal Study of Adolescent Health, 2206 female, 2237 males who had romantic partnership between Wave 1 and 2  Wave1=1995  Wave 2=1996  Participation rate=not reported  Attrition rate=not reported | Physical and verbal, modified CTS, assessed incident IPV between Waves 1 and 2.  Physical/verbal: “call you names, insult you, or treat you disrespectfully in front of others, swear at you, threaten you with violence, push or shove you, and throw something at you that could hurt you”. Also asked about timing of initiation, constructed measure of incident IPV based on this. | Depression: changes in CES-D score between Waves1 and 2, past week.  Suicidal behavior, not further specified | IPV and incident depression  Female: **β=0.18 (0.10-0.26), p<0.005**  Male: β=0.08 (0.00-0.16)  IPV and incident suicide attempt  Female: **β=0.12 (0.02-0.22)**  Male: β=-0.00 (-0.06-0.06) | Sociodemographic factors, highest severity of  abuse prior to Wave 1, number of intimate partners between Waves 1 and 2,  time elapsed between Waves 1 and 2, and baseline risk behaviour involvement | IPV assessed only at one time point, timing of abuse constructed based on wave 2 reports. Depression and suicide measured at waves 1 and 2. |
| Salazar[[24](#_ENREF_24)]  Nicaragua, Leon cohort.  370 women, pregnant at baseline.  Wave 1=2002-2003  Wave 2=2007  Participation rate=not reported  Attrition rate=17% | Physical and/or sexual IPV, wave 1 and wave 2. WHO instrument. | Depression: SRQ-20, with scores of 7 or more indicative of probable emotional distress | Depressive symptoms at baseline, odds of continued abuse at follow-up (ie. incident cases):  **OR=2.42 (1.46-4.02), p=0.002**  IPV at baseline, odds of depressive symptoms at follow-up:  **OR=2.01 (1.08-3.78), p=0.0178** | Unadjusted | Calculated from information in paper. For incident IPV, using ‘continued abuse’ as incident IPV and ‘ending abuse’ and never abused as no incident cases.  For depressive symptoms at follow-up, comparison is between ‘ending abuse’ and never abused so all IPV is at baseline. |
| Suglia[[34](#_ENREF_34)]  1834 women, recent birth at baseline  Wave 1=1996-1997  Wave 2=1997-1998  Wave 3=1999-2000  Participation rate=not reported  Attrition rate=57.1% | Physical at wave 1, physical and/or sexual at wave 2, 3.  Physical, wave 1, from baby’s father: “How often does he hit or slap you when he is angry?”  Physical, wave 2,3, from baby’s father or current partner: “How often does he slap or kick you? How often does he hit you with a fist or object that could hurt you? Were you ever cut or bruised or seriously hurt in a fight?”  Sexual, wave 2, 3, from baby’s father or current partner: “How often does he try to make you have sex or do sexual things that you want to do?” | Depression:  CIDI-SF screening instrument, at wave 2, 3, past year time period. | IPV before wave 1 and incident depression:  aOR=1.09(0.6-1.9)  IPV between wave 2 and 3 and depression between wave 2 and 3:  aOR=1.59(0.9-2.7)  IPV at wave 1,2,3 and depression between wave 2 and 3:  aOR=1.12(0.5-2.5) | Age, race, education, marital status, housing deterioration, housing disarray, housing instability, economic hardship | Variables possibly on causal pathway controlled (IPV could cause housingvariables) |
| Taft [[21](#_ENREF_21)]  Australia, Australian Longitudinal Study of Women’s Health younger cohort, 9683 women aged 18-23 at Wave 1  Wave 1=1996  Wave 2=2000  Participation rate=41.1%  Attrition rate= 34.5% | Physical and/or sexual IPV, either new violence at Wave 2 (versus never), or violence at wave 1 only or wave 1 and 2.    “In the last twelve months, have you been pushed, grabbed, shoved, kicked or hit? Have you been forced to take part in unwanted sexual activity?”  “Have you ever been in a violent relationship with a partner/spouse?” | Depression: CES-D 10, wave 2.  Single question on ‘ever been told by a doctor or nurse that you are depressed’ in Wave 2, in the past 4 years and more than 4 years ago (used to control for Wave 1 depression). | IPV wave 2-Depression at wave2:  **aOR=2.12 (1.69 – 2.65)**  IPV at wave 1 or 2 vs never violence-depression wave 2 **aOR=2.06 (1.74 – 2.43)** | Termination violence, depression in 1996, number of children, marital status, age, education level,  occupation, health insurance status, country of birth, area of residence, state of residence and Aboriginal or Torres Strait identity | IPV at wave 1-depression wave 2 included in meta-analysis-longitudinal. For other one more chance that depression actually occurred before new IPV. |
| Zlotnick [[28](#_ENREF_28)]  USA, National Survey of Families and Households. 3104 married or cohabiting women, aged 19+  Wave 1=1987-1988  Wave 2=1992-1994  Participation rate=not reported  Attrition rate=23% | Physical IPV:  “During the past year, how many fights with your partner resulted in him/her hitting, shoving, or throwing things at you? Have you ever been cut, bruised, or seriously injured in a fight with your partner?” | Depression: CES-D, past week symptoms, assessed at Wave 2 | **β=6.96.*p* = 0.003** | Age, Wave 1 depression | N with violence at wave1=148. IPV only assessed in those who were married/cohabiting at the time of the interview, so we don’t know if women who left their partners continued to experience IPV (46% of all women with IPV at wave 1) |

CSA is childhood sexual abuse; CTS is Conflict Tactics Scale; WHO is World Health Organisation; AAS is Abuse Assessment Screen; CES-D is Center for Epidemiologic Studies-Depression, BSI is Brief Symptom Inventory; BDI is Beck Depression Inventory, CIS-R is Clinical Interview Schedule-Revised; DICA-R-A is Diagnostic Interview for Children and Adolescents-Revised-Adolescents; SVAWS is Severity of Violence Against Women Scales; CIDI-SF is Composite International Diagnostic Interview-Short Form; SRQ-20 is Self-Report Questionnaire-20; MINI is Mini-International Neuropsychiatric Interview Interview
